# Current status and future perspective of genomics-assisted breeding in wheat (*Triticum aestivum* L.)

**DOI:** 10.1270/jsbbs.25059

**Published:** 2026-02-21

**Authors:** Tsuyoshi Tanaka, Fuminori Kobayashi, Goro Ishikawa

**Affiliations:** 1 Research Center for Advanced Analysis, NARO, 2-1-2 Kannondai, Tsukuba, Ibaraki 305-8602, Japan; 2 Institute of Crop Science, NARO, 2-1-2 Kannondai, Tsukuba, Ibaraki 305-8518, Japan

**Keywords:** marker-assisted selection, genetic analysis, genomic selection, hexaploid, genome sequencing, genotyping, breeding strategy

## Abstract

This review summarizes the current status and future prospects of using genomic information for wheat breeding. Wheat has the largest genome among all major crops (~16 Gb), thus requiring a sophisticated approach to collect and utilize genomic information compared to other crops. In this review, we first describe the conventional methods of marker-assisted selection in wheat breeding. We discuss results from studies using DNA markers, such as those breaking the tight linkage between disease resistance and undesired quality traits. Although marker-assisted selection has achieved some success, breeding efficiency cannot be easily improved using this technique alone because several important traits, such as yield, are governed by a large number of genes. Recently-developed tools for genetic analysis, such as next-generation sequencing, are being increasingly used in wheat research. Therefore, we outline the history and current status of wheat genome resources, including reference genome sequencing, databases, analysis tools, and genotyping platforms. Further, we discuss the prospects for wheat breeding based on these resources. This review highlights the importance of incorporating new technologies to breed wheat varieties with high yield and quality.

## Introduction

Wheat (*Triticum aestivum* L.) is the most widely cultivated crop in the world, contributing to approximately 18% of the total caloric intake globally, and represents the largest proportion of food sources consumed by humans (FAOSTAT, Food Balances 2022 data https://www.fao.org/home/en). In Japan, wheat accounts for 13% of the total caloric intake, second only to rice (20%; FAOSTAT). Wheat is deeply rooted in Japanese food culture and is processed into a wide variety of food products, such as udon (Japanese salted noodles), ramen (Chinese alkaline noodles), bread, and traditional pastries. Since the self-sufficiency rate of wheat in Japan is approximately 15%, the Japanese government is attempting to expand domestic wheat production from 1.09 million tons in 2023 to 1.37 million tons by 2030 to ensure food security (Ministry of Agriculture, Forestry and Fisheries, Japan, https://www.maff.go.jp/j/syouan/keikaku/soukatu/mugi_kanren.html (in Japanese)). To achieve this, it is essential to develop climate-resilient varieties with stable yields, in addition to increasing yields. Climate change has affected the primary wheat production areas, including Europe, Australia, and the US, with changes in growth rates occurring due to rising temperatures and disease outbreaks. Thus, wheat breeders worldwide are striving to develop improved varieties by fine-tuning genetically-complex yield and end-use quality, while maintaining yield stability and regional adaptation to specific biotic and abiotic stresses.

Selection is the process of accumulating useful genes, thus understanding the genetics of target traits is critical for improving breeding efficiency. Studies have been conducted to link traits to genetic information in various crop species, and these results have been applied in breeding programs. However, wheat has lagged behind other species, primarily because of the challenges of assembling large (~16 Gb) (Arumuganathan and Earle 1991), hexaploid, and complex genomes that contain more than 85% repetitive DNA (Appels *et al.* 2018). Recent advances in sequencing technologies and analysis tools have paved the way for the use of genomic information in wheat breeding. Fully annotated and ordered genome sequences, including regulatory sequences and genome diversity information, have promoted the development of systematic and time-efficient approaches for selecting and understanding important traits.

Several comprehensive reviews have discussed wheat genetics and breeding using the latest technologies (Paux *et al.* 2022, Sun *et al.* 2023a, Yao *et al.* 2025). Therefore, in this review, we focus on the use of genomic information for practical breeding programs, particularly in Japan, where wheat varieties are primarily being developed by public institutions. The main themes of this review are marker-assisted selection (MAS) of major effect genes, genome sequencing by international consortia, global databases, genotyping platforms, and genome-wide marker selection. Furthermore, we discuss the prospects for future wheat breeding by combining these technologies. Globally, wheat breeding is conducted by seed companies of various sizes and public institutions, each operating at different scales. This review elaborates the most appropriate methods for each scale of operation, thus this review will be useful not only in Japan but also in breeding programs in worldwide.

## Functional markers for wheat breeding

Functional or diagnostic markers that can detect DNA polymorphisms associated with phenotypes have been developed worldwide. The functional markers utilized in wheat breeding programs are summarized in Table 1. Wheat has three homoeologous genes that perform similar functions in a compensatory manner, making it difficult to identify relationships between genes and traits. However, more than 170 genes have been isolated in wheat (Korchanová *et al.* 2025), and decades of research have identified approximately 70 markers that are available for breeding purposes. In Japanese breeding programs, MAS for quality trait genes, such as those related to amylose content, dough properties, and flour color, began around the year 2000. This was followed by MAS for disease-resistant, pre-harvest sprouting resistance, and other agronomically important genes.

The first example of a marker used in a Japanese breeding program was the *Wx* gene (*Wx-A1*, *Wx-B1*, and *Wx-D1*), which is involved in determining the amylose content of wheat flour (Nakamura *et al.* 2002, Saito *et al.* 2009). Its effectiveness as an alternative to the tedious measurement of amylose content was confirmed, leading to the establishment of MAS in wheat breeding. Markers for the *Wx* genes have also been used to develop waxy wheat varieties. Subsequently, *Wx* gene markers were combined with markers for the *SSIIa* genes (*SSIIa-A1*, *SSIIa-B1*, and *SSIIa-D1*) (Shimbata *et al.* 2005), which encode amylopectin synthase, leading to the development of sweet wheat and breeding lines with reduced starch retrogradation (Inokuma *et al.* 2016, Nakamura *et al.* 2006). Owing to its large selection effect, DNA markers for *Glu-1* genes (*Glu-A1*, *Glu-B1*, and *Glu-D1*), which encode a high-molecular-weight glutenin subunit and are involved in dough properties, have been developed and used in MAS (Ahmad 2000, Butow *et al.* 2004, Ishikawa and Nakamura 2007). Markers for low-molecular-weight glutenin subunit genes have also been developed (Ikeda *et al.* 2006, Maruyama-Funatsuki *et al.* 2005, Zhang *et al.* 2004) and are used along with those for high-molecular-weight glutenin to select candidates with suitable dough properties. Because quality tests typically require large amounts of grain, DNA markers for genes related to quality traits can be used to identify inferior breeding lines earlier, potentially saving resources (Kiszonas and Morris 2018). These DNA markers have been utilized in the development of high-quality wheat cultivars. For improved bread-making quality, the alleles *Glu-D1d* (HMW-GS), *Glu-B3h* (LMW-GS), and *Pinb-D1c* (associated with hard grain texture) were successfully combined in the cultivar ‘Setokirara’ (Takata *et al.* 2017). In the breeding of ‘Kinuakari’, four glutenin subunit alleles—*Glu-A1b*, *Glu-B1b*, *Glu-A3d*, and *Glu-B3g*—along with the *Wx-B1* deletion allele, were introduced to enhance the quality of Japanese white salted noodles (Wheat Cultivar “Kinuakari” Breeding Group 2022).

The wheat cultivar ‘Tamaizumi R’ was also developed with MAS to improve agronomic traits while maintaining the quality of ‘Tamaizumi’, which is a hard white wheat cultivar with a high protein content (Kiribuchi-Otobe *et al.* 2019). During its breeding, a resistance QTL, *Q.Ymym*, and a deletion mutation of the *TaABA8’OH1-D* gene were introduced, improving resistance to wheat yellow mosaic disease and pre-harvest sprouting, respectively (Chono *et al.* 2013, Kojima *et al.* 2015, Nishio *et al.* 2010). ‘Tamaizumi R’ was adopted as a recommended variety in Mie Prefecture and Gifu Prefecture, Japan. In other countries, MAS has been actively applied to introduce disease resistance genes into wheat cultivars. A notable example is the utilization of the *Lr34* gene, which confers broad-spectrum resistance to multiple pathogens (Krattinger *et al.* 2009). In India, wheat varieties incorporating *Lr34* have been successfully developed, demonstrating its effectiveness in enhancing disease resistance under field conditions (Saini *et al.* 2024).

Despite the development of a large number of DNA markers for wheat breeding, as summarized in Table 1, their practical availability and utilization within Japanese breeding programs remain limited. This is partly because their effectiveness has not been confirmed in domestic cultivars. In addition, breeding goals vary by region, and wheat’s polyploidy complicates marker interpretation due to homoeologous genes. Furthermore, the application of MAS is limited to traits with relatively simple heritability. Traits such as yield and processing quality are inherited in a complex manner involving multiple genes. Therefore, it is necessary to improve these traits using genome-wide information.

## Characteristics of the wheat genome

The construction of wheat genome sequences is crucial for enhancing and promoting the genetic dissection of important traits in wheat breeding programs. Sequencing the genome of the wheat cultivar ‘Chinese Spring’ began in 2005 with international collaboration (for more information, see the IWGSC website, https://www.wheatgenome.org/about). Wheat is characterized by a large genome size compared to other major crops, caused by more than 85% of repetitive regions and polyploidy (Appels *et al.* 2018, Aury *et al.* 2022, Wang *et al.* 2025). Specifically, the presence of the A, B, and D genomes makes it difficult to accurately assemble genome sequences. Therefore, sequencing has been performed on a chromosome-by-chromosome basis using chromosome sorting technology (Kubaláková *et al.* 2005). The physical map of chromosome 3B, the largest chromosome, was published in 2008 (Paux *et al.* 2008). This chromosome was separated from other chromosomes by flow cytometry (Vrána *et al.* 2000), demonstrating the effectiveness of this technique. Notably, this success went down in history as a milestone in high-quality genome sequencing of large-genome crops. Sequencing of chromosome 6B was performed by a Japanese research consortium, comprising Yokohama City University, Kyoto University, Nisshin Flour Milling Inc., the Institute of Experimental Botany (Czech Republic), and the National Institute of Agrobiological Sciences. Survey sequences and physical maps of chromosome 6B were published in 2014 and 2015, respectively (Kobayashi *et al.* 2015, Tanaka *et al.* 2014). Although these results only represent a small portion of the whole genome, they led to the identification of a large number of DNA markers that could be used for genetic research (Kobayashi *et al.* 2021). After the release of the first survey sequences of the ‘Chinese Spring’ genome (Eversole *et al.* 2014), the refinement of the genome sequence continued. Chromosome-level assemblies were published in 2018 (Appels *et al.* 2018; RefSeq v.1) and were updated in 2021 (Zhu *et al.* 2021; RefSeq v.2) with the help of new genome sequencing and assembling technologies, such as DeNovoMAGIC^TM^, optical mapping, and long-read next-generation sequencing (NGS). Notably, genome sequences and annotations are continuously being updated. Recently, the genome assembly and comprehensive annotation was updated by different groups (Liu *et al.* 2025, Wang *et al.* 2025).

## Examples of utilizing genome information in breeding

The development of wheat genome information has accelerated the reverse genetic identification of agronomically important genes by leveraging insights and methodologies established in other crop species (Korchanová *et al.* 2025). Comparative genomics approaches leveraging rice gene information have facilitated the identification of wheat orthologs such as *TaGW2-6A* and *TaTGW6-A1* (Hanif *et al.* 2016, Su *et al.* 2011). Advances in genome information across wheat and its wild relatives have enabled the identification and isolation of agronomically important genes, including those associated with disease resistance such as *Rmg8* (Asuke *et al.* 2024). These genes are used as functional markers to enhance breeding efficiency (Table 1).

**Fig. 1.**
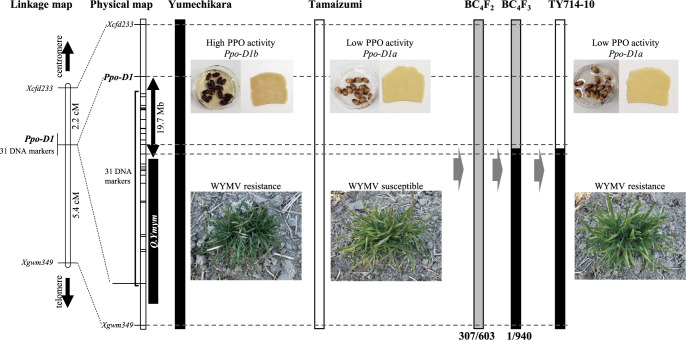
Development of new breeding materials by combining genome sequence information and MAS: breaking the tight linkage between wheat yellow mosaic disease resistance *Q.Ymym* and high activity allele of PPO gene, *Ppo-D1b* (Compiled from Kobayashi *et al.* 2020). The graphical genotypes of ‘Yumechikara’, ‘Tamaizumi’, their BC_4_F_2_ and BC_4_F_3_ progeny, and TY714-10 indicate the allelic composition at *Ppo-D1* and *Q.Ymym*. ‘Yumechikara’-type is black, ‘Tamaizumi’-type is white, and heterozygous is grey. A recombinant line carrying heterozygous *Ppo-D1* and *Q.Ymym* was screened in 940 BC_4_F_3_, from which ‘TY714-10’ with *Ppo-D1a* and *Q.Ymym* was obtained. The three photos for each variety show the result of the following test: the top left is the phenol reaction test, the top right is the raw noodle discoloration test, and the bottom is the WYMV resistance test.

Genome sequence information reveals the order of genes along a chromosome, enabling the precise selection of markers for specific purposes, such as the solution of linkage drug. This contributes to more efficient MAS and enables the development of new breeding materials that were previously rarely obtainable. One such example is the separation of the tight linkage between the yellow mosaic disease resistance gene (*Q.Ymym*) and the high-activity allele of the polyphenol oxidase gene (*Ppo-D1b*) (Fig. 1) (Kobayashi *et al.* 2020). The linkage map constructed by Kobayashi *et al.* (2020) showed that *Ppo-D1* and 31 DNA markers linked to *Q.Ymym* are present at the same locus. The simultaneous introduction of *Q.Ymym* and *Ppo-D1b* has been challenging in breeding. Genome sequence information further revealed the physical positions of *Ppo-D1*, 31 DNA markers, and *Q.Ymym*, leading to the successful development of new breeding material that broke the tight linkage between the two genes, thereby resolving the problem.

## Genome data comparison and pan-genome

**Fig. 2.**
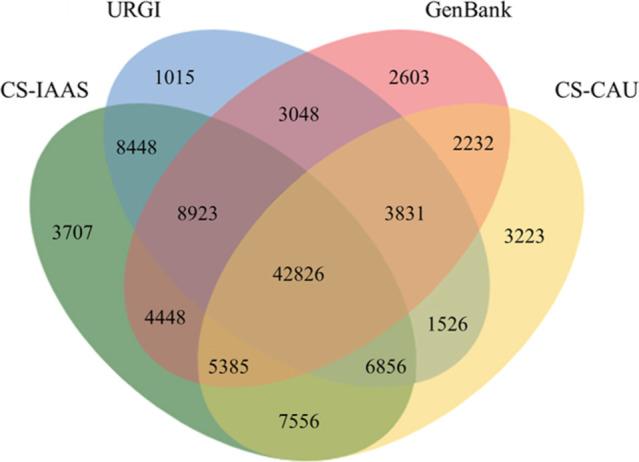
Comparison of annotated genes in URGI (Chinese Spring RefSeq v.2.1) (Zhu *et al.* 2021), CS-IAAS (Liu *et al.* 2025), GenBank (GCF_018294505.1), and CS-CAU (Sun *et al.* 2023a). Clustering was performed by OrthoVenn3 with the OrthoMCL algorithm with default parameters (Li *et al.* 2003, Sun *et al.* 2023b).

While updated genome sequences provide more accurate information, it is important to understand the differences in content between datasets, such as annotation data, gene IDs, and physical positions. For example, the four genomes derived from URGI (Chinese Spring RefSeq v.2.1) (Zhu *et al.* 2021), CS-IAAS (Liu *et al.* 2025), GenBank (GCF_018294505.1), and CS-CAU (Sun *et al.* 2023a), show differences in total assembly length, chromosome lengths, and the number of predicted genes (Table 2, Supplemental Table 1). OrthoMCL (Li *et al.* 2003) clustering of four annotation datasets using OrthoVenn3 with the default setting (Sun *et al.* 2023b) showed the existence of database (DB)-specific gene families (Fig. 2). Moreover, these annotation data use different gene IDs, requiring the creation of ID lists for precise comparison. For example, one of the alpha/beta gliadin genes on Chr6A was TraesCS6A03G0110100 in URGI, CSIAAS6AG0160000HC.mRNA1 in CS-IAAS, LOC123131794 (gene ID), and XP_044407408.1 (protein ID) in GenBank. Therefore, it is essential to specify the source and dataset version for analyses using genome data to avoid confusion. The quality and accuracy of genome sequences and their annotation data cannot currently be evaluated. One of the best ways to select reference genomes may be to consider the frequency with which the data are updated.

The genome sequencing of wheat accessions other than the cv. ‘Chinese Spring’ was achieved by advancements in NGS technologies and assembly tools. For example, in 2020, the 10+ Wheat Genome Project released 10 chromosome-level genome assemblies, including the Japanese representative cultivar ‘Norin 61’, and five draft genome sequences (Walkowiak *et al.* 2020). Genome sequences of other cultivars, such as cv. ‘Fielder’, ‘Renan’, ‘Kariega’, ‘Aikang 58’ and ‘Chunmai104’ are also publicly available (Athiyannan *et al.* 2022, Aury *et al.* 2022, Jia *et al.* 2023, Liu *et al.* 2024, Sato *et al.* 2021), and another 17 pan-genome sequences have been published (Jiao *et al.* 2025). Long-read NGS data revealed long repetitive regions, including telomeric and centromeric regions, and distinguished highly similar sequences between homoeologous and paralogous regions. This data also enables the detection of large structural variations at the chromosomal level, which can be useful information for breeding (Jiao *et al.* 2025). In contrast to genome resequencing studies, direct comparison of *de novo* genome sequences can reveal differences in genome structure and haplotypes (Liu *et al.* 2025). Pan-genome data provides a comprehensive set of wheat genes. Even if some genes are absent in individual cultivars, they may be present in others, allowing the pan-genome data to compensate for these differences. This also indicates that the selection of reference genome sequences is important for appropriate genome analysis and depends on the purpose of the study.

## Databases and analysis tools

Several wheat genomics databases are available. Unlike the primary database, which contains original and unprocessed biological data and includes the DNA Database of Japan (DDBJ), the European Molecular Biology Laboratory (EMBL), and GenBank at the National Center for Biotechnology Information (NCBI), secondary databases provide curated and analyzed data derived from primary sources. The most fundamental secondary databases for wheat include genome databases such as URGI, which hosts the IWGSC genome assembly, and CAU, which provides the CS-CAU genome assembly (Table 2). These databases offer assembled genome sequences and associated annotation data. The increase in wheat genome data has resulted in a fragmented landscape of genome databases, making it difficult for researchers to find relevant datasets. To address this inconvenience, portal databases providing access to genome sequences have been developed, such as EnsemblPlants (Yates *et al.* 2022), GrainGenes (Yao *et al.* 2022), and PlantGARDEN (Ichihara *et al.* 2023). While portal databases are convenient for searching and downloading appropriate data from multiple genome resources, it is important to consider the version of the data used. The synchronization of data between secondary and original databases depends on the database administrators, which can cause time lags, resulting in confusion due to different data versions.

**Fig. 3.**
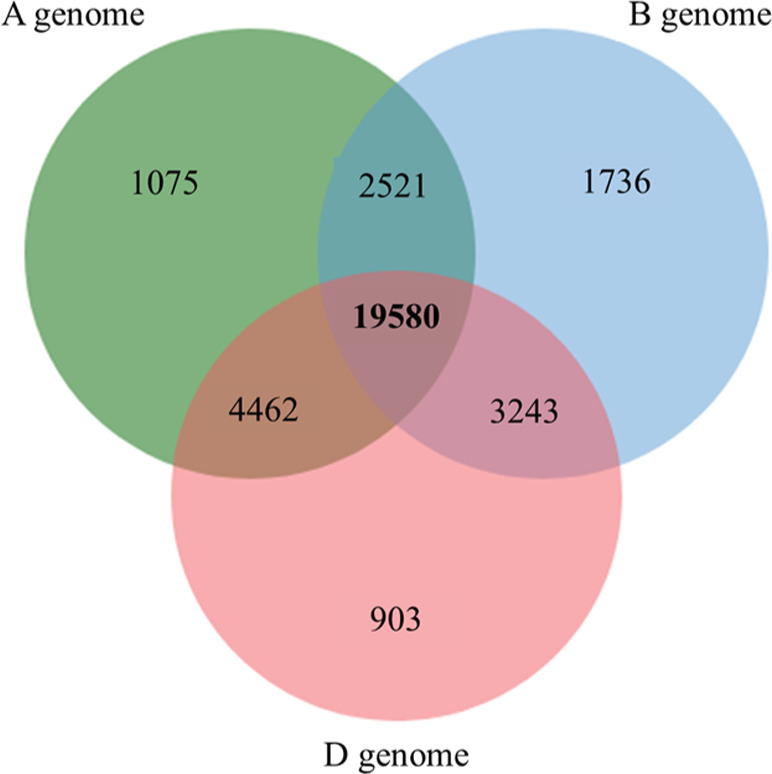
Venn diagram for the ortholog clustering of genes from A, B, and D genomes. Gene annotations were obtained from Wang *et al.* (2025). Clustering of high-similarity genes was performed by OrthoVenn3 with the OrthoMCL algorithm with default parameters (Li *et al.* 2003, Sun *et al.* 2023b).

While every database provides various analysis tools to facilitate efficient data retrieval, with the Basic Local Alignment Search Tool (BLAST) being the most widely used tool (Altschul *et al.* 1990), there are many online genome analysis services and tools for the particular purposes, and genome browsers to visualize results of genome analysis (Supplemental Table 2). For instance, web services are available for *in silico* analyses of genes in detail. Genome-wide SNPs with 30,217 loci which were obtained from the Japanese wheat core collection (Kobayashi *et al.* 2016, Mizuno *et al.* 2024) were compiled in TASUKE+ (Kumagai *et al.* 2019). In case of gene ontology (GO) analysis, users upload a tab-delimited text file containing the list of genes and GOs on GOslim in AgBase and the summarized results are returned in three categories. Analysis using the CS-CAU (Wang *et al.* 2025) gene sets from the wheat cultivar ‘Chinese Spring’ revealed that many homoeologs and paralogs are distributed in the A, B, and D genomes using OrthoVenn3 service (Fig. 3) (Sun *et al.* 2023b), highlighting the need to distinguish mutations between homoeologs and polymorphisms between cultivars in marker construction.

For genome-wide association studies, phenotypic information is as essential as genomic data. For example, WheatOmics 1.0, which provides comprehensive multiple-omics data for wheat (http://202.194.139.32/) (Ma *et al.* 2021), while National Bioresource Project (NBRP) KOMUGI (https://shigen.nig.ac.jp/wheat/komugi/), a database of wheat genetic resources, offers phenotypic and marker information. In particular, NBRP KOMUGI allows users to search for appropriate samples for their research based on phenotypes.

## Genome-wide genotyping platform

Genome-wide DNA polymorphism information has been used for diversity analyses, genetic analyses, and trait prediction. In recent years, owing to the decreasing costs of sequencing (https://www.genome.gov/about-genomics/fact-sheets/DNA-Sequencing-Costs-Data), whole-genome sequencing has become the most effective method for obtaining genome-wide polymorphisms in crops with small genome sizes, such as rice (Huang *et al.* 2010, Wang *et al.* 2018). An international framework led by groups in the UK, China, and other countries has reported whole-genome resequencing of approximately 1,000 landraces of wheat, even those with large genome sizes (Cheng *et al.* 2024). However, whole-genome sequencing of wheat is expensive in terms of data acquisition and analysis. In genetic analysis and breeding selection, where genome-wide polymorphism information is required for many samples, it is impractical to obtain polymorphism information using whole-genome sequencing.

Techniques, such as amplified fragment length polymorphism (AFLP), simple sequence repeat (SSR), and insertion site-based polymorphism (ISBP), have been developed to identify genome-wide DNA polymorphisms (Paux *et al.* 2010, Röder *et al.* 1998, Schwarz *et al.* 2000, Somers *et al.* 2004). Over the past several decades, general genotyping methods have shifted from gel-based to fluorescence-based systems. Gel-based systems detect size differences in PCR products, whereas fluorescence-based systems detect single-nucleotide polymorphisms (SNPs). The fluorescence-based SNP detection system, such as Komparative Allele Specific PCR (KASP) (Rasheed *et al.* 2016, Semagn *et al.* 2014), is compatible with automation and is suitable for breeding selection where large numbers of samples need to be examined in a short period of time. However, genome-wide genotyping using these techniques is time-consuming and laborious. Subsequently, SNP array technology has been established. Briefly, this array aligns DNA sequences, called probes, on a slide, allowing the simultaneous analysis of many SNPs through differences in fluorescence intensity (LaFramboise 2009). As an alternative approach using NGS, the genotyping-by-sequencing (GBS) method has been developed to sequence several samples simultaneously by adding an identifier, called an index (Elshire *et al.* 2011). Currently, SNP arrays and NGS are the primary methods used for genome-wide genotyping of multiple wheat samples.

Wheat SNP arrays developed to date are listed in Table 3. Earlier arrays were developed using SNP information derived from exome sequences (Cavanagh *et al.* 2013, Wang *et al.* 2014). These arrays were revolutionary and have been widely used for various genetic analyses. However, they exhibited intra-chromosomal probe bias and uneven probe distribution among the three genomes. Subsequently, an Axiom array applicable to a wide variety of materials, including wild species, was developed (Winfield *et al.* 2016). Since then, several groups have produced improved versions of SNP arrays (Allen *et al.* 2017, Cui *et al.* 2017, Kitt *et al.* 2021, Rimbert *et al.* 2018). Sun *et al.* (2020) compared several arrays and concluded that the wheat 660 K array contained the highest percentage of genome-specific SNPs with reliable physical positions. Furthermore, the SNPs in the array were almost evenly distributed across the entire genome. More recently, a novel Axiom array, the ‘*Triticum aestivum* Next Generation’ array (TaNG), largely derived from whole-genome skim sequencing of more than 300 lines, was developed and showed improved performance compared to the 35 K chip (Burridge *et al.* 2024), highlighting the superiority of the data in newer arrays. From the perspective of data reproducibility and experimental simplicity, the SNP array is the most suitable tool for wheat genetic analysis and is constantly being improved.

NGS methods offer greater flexibility compared to array-based methods. A wide range of NGS platforms is available, with outputs ranging from 5 Gb to 8 Tb, allowing researchers to choose sequencers depending on the amount of data required. When a large number of samples need to be analyzed simultaneously, a portion of the genome in each sample is sequenced to reduce costs. NGS consists of random and targeted methods. Random methods such as RAD-seq and MIG-seq use restriction enzymes and common primers, respectively, to enrich and sequence portions of the genome (Nishimura *et al.* 2022, Poland *et al.* 2012, Suyama and Matsuki 2015). GRAS-Di is also widely used as a method for randomly sequencing portions of the genome (Enoki and Takeuchi 2018). Targeted methods include sequence capture and amplicon sequencing, which enrich target sequences using probes and primers containing known polymorphic sites (Bernardo *et al.* 2020, Burridge *et al.* 2022, Ishikawa *et al.* 2018). The quality and quantity of data required depend on the research objective, and this flexibility is the main appeal of NGS.

Changes in detection methods have led to alterations in the use of genetic markers. Initially, MAS was limited by the number of samples that can be analyzed. Therefore, backcross breeding introducing a single gene was the most powerful approach that uses DNA marker effectively (Ribaut and Hoisington 1998). Recently, high-throughput analyses have made it possible to select individuals from large populations at early breeding stages. For example, if essential disease resistance or quality genes are present, selection efficiency can be enhanced by excluding individuals that do not possess these genes at an early stage. The various methods for obtaining wheat genomic information and characteristics are summarized in Table 4. The best method for obtaining genome-wide genotypes depends on the purpose of the analysis and the type of material used. Acceptable costs vary significantly between genetic analysis and breeding selection. In genetic studies involving genetic resources or panels of parental cultivars and leading varieties, it may be worthwhile to invest in genome-wide polymorphism data. However, when several inferior individuals are discarded, as is the case in breeding selection, the cost-effectiveness of genotyping these individuals must be carefully considered. Sequencing methods also need to be selected based on the material and research purpose (Table 5). Random methods are suitable for new materials with limited genomic information, whereas targeted methods are suitable for breeding materials. Skim sequencing, a technique that sequences several samples with very low coverage and imputes missing data, is another effective genotyping approach. However, this method should only be used for materials with known relationships. Therefore, in wheat, it is important to use different methods to obtain genome-wide genotypes depending on the material and purpose (Table 5).

## Future perspective

In the last decade, the quality and quantity of genomic information on wheat have been greatly enriched, enabling the detection of many polymorphic sites (genomic regions) related to disease resistance, quality, and yield (see databases such as GrainGenes, https://testing.graingenes.org/GG3/; URGI, https://wheat-urgi.versailles.inra.fr/; and WheatQTLdb V2.0, http://www.wheatqtldb.net/)). This genetic information has been used for gene identification and MAS breeding. Genome-wide polymorphism information has also been used to create models to predict trait values (Meuwissen *et al.* 2001). These models allow for selection based on the predicted trait values without investigating the actual trait values (Crossa *et al.* 2014, Heffner *et al.* 2009, 2010, Juliana *et al.* 2020). Furthermore, this technology makes it possible to predict the genotypes of progenies derived from any given cross-combination and further predict trait values (Hamazaki and Iwata 2024, Iwata *et al.* 2013). Therefore, breeding efficiency can be increased by predicting cross combinations with a higher probability of producing candidates with the desired trait values.

In a seed company in Australia, a generation acceleration method called “speed breeding” and “speed vernalization” (Cha *et al.* 2022, Guo 2022, Watson *et al.* 2018) was implemented in practical breeding programs. After generating F_5_ and F_6_ generations, all materials were genotyped on an array-based platform and analyzed as described above. Acquiring genotypic information on an array-based platform using tens of thousands of materials is cost-prohibitive. However, the company reduced costs using a custom array containing both wheat and barley probes on the same slide. In another study, a large seed company in Europe reduced procurement costs by using the same genotyping platform for all crops handled, including maize, sugar beets, wheat, barley, rye, and sunflowers (personal communication).

**Fig. 4.**
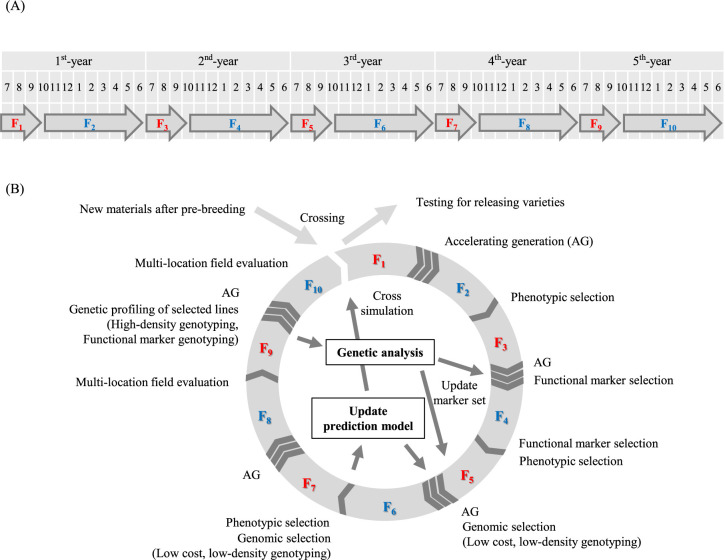
A schematic diagram of the proposed breeding process, the “Breeding Ring”, which consists of accelerating generation, marker-assisted selection, genomics selection, and genetic analysis. (A) Cultivation calendar from F_1_ to F_10_ in an area where the wheat growing season is eight months or less, e.g. western region of Japan. (B) Work items carried out at each generation. It is important to carry out selection while advancing genetic analysis and model updating throughout the breeding process.

Currently, array-based platforms are considered the simplest and most stable way to obtain data for genotyping many wheat samples. However, breeding and selection may not always be optimal owing to the excessive cost and amount of data involved. Considering these issues, we propose a new breeding process for wheat, called the “Breeding Ring” (Fig. 4), which combines accelerated generation, functional marker selection, genomic selection, and genetic analysis in a circular manner. In this method, odd-numbered filial generations are subjected to accelerated generation after crossing, and only even-numbered generations are evaluated for traits in the field. Selected F_10_ lines can be obtained within five years (Fig. 4A). Field evaluations alone take too much time, and accelerated generation alone cannot ensure accurate selection. It is crucial to combine both approaches across all generations. The breeding process is often represented as a straight line, from crossing to the development of a variety. However, most of the selected lines are not released as varieties but become parental materials for the next step of breeding, which is a major driving force for improvement. Therefore, we believe that the breeding process is more accurately represented by a circle (Fig. 4B). During this process, negative selection (removal of inferior individuals) is performed in the F_3_ and F_4_ generations using a small set of functional markers. These selection steps reduce the number of individuals undergoing genome-wide genotyping, leading to cost reduction. In the F_5_ and F_6_ generations, genomic selection is performed using a low-density, low-cost genotyping platform. These steps enable the selection of complex traits that were previously only selectable in later generations, making it important for accelerating the pace of improvement. By accumulating trait values and genotypes of the F_6_ generation, the trait prediction model for genomic selection can be updated. Trait prediction relies on the relationship between the population used for model construction and the target population for prediction. By updating the model with data from the generation, the latest model can always be applied to the next batch of material. In the F_8_ generation, an array-based platform is applied to obtain high-density SNP data. Genome-wide association studies (GWAS) can be performed on trait values and genotypes from the F_8_ generation to identify novel genetic factors. Polymorphic DNA sites significantly associated with traits of interest are added as targets for genotyping. Since accelerated generation requires a cultivation period of 3–4 months, this method can be applied in areas where the wheat growing season is eight months or less, for example, in western Japan. For wheat growing periods longer than 8 months, we also propose two other processes (Supplemental Figs. 1, 2).

**Fig. 5.**
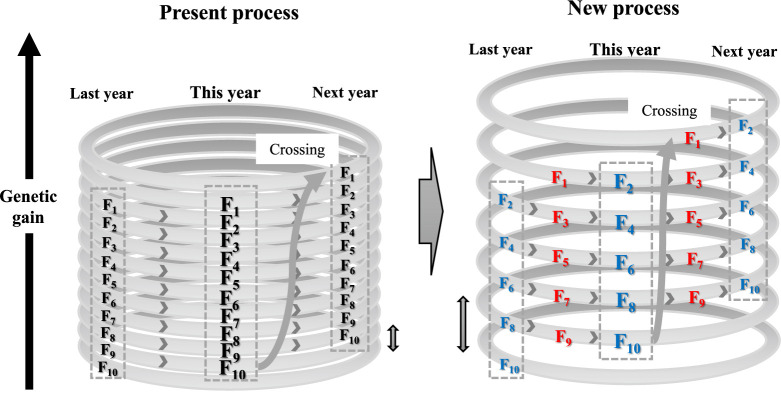
Schematic diagram showing the multi-layered nature of the “Breeding Rings” in actual breeding. The new process increases the range of genetic gain between rings and improves the speed of improvement.

The key aspect of this breeding process is that genetic analysis is conducted using materials undergoing breeding and selection. In the past, the materials used in genetic analyses often differed from those used in breeding. This is the main reason why the detected genes are not used for breeding. The proposed breeding process uses the latest breeding materials for genetic analysis, enabling the identification of new genetic factors related to traits that have not been fully selected in the current breeding population. In actual breeding, new crosses are created annually, resulting in a multi-layered structure referred to as “Breeding Rings” (Fig. 5). This proposed method is expected to increase genetic gain per cycle and significantly increase breeding efficiency compared to conventional methods. To integrate breeding with genetic analysis, it is necessary to establish a system for efficiently collecting and analyzing the vast amounts of data generated per “Breeding Ring”. Currently, the lack of a data management system suitable for implementing this process is a major obstacle, and its development is urgently needed. Advances in genome analysis technology have eliminated technical barriers to genomics-assisted breeding, even for wheat, which has a large and complex genome. The challenge lies in integrating these technologies into the breeding process while accounting for their cost and effectiveness. Although the methods presented in this review are currently considered optimal, they should be updated as the related technologies continue to advance.

## Conclusion

Recent advances in genome analysis technology have eliminated most technical barriers to genetic analysis and breeding selection, even in wheat, which has a large and complex genome. However, cost remains a major constraint in breeding programs involving large numbers of individuals, and it is not always possible to apply these methods to other major crops, such as rice and soybean. This review summarizes the latest information on known wheat genes and functional gene markers, the status of the reference genome and wheat genome characteristics, global wheat databases and tools, and genotyping platforms using SNP arrays and next-generation sequencers. In addition, we propose a novel breeding framework called “Breeding Rings”. The most important aspect for implementing genomic information in breeding is bridging the gap between genetic analysis and breeding selection materials. The proposed breeding process can help in this regard and is expected to evolve alongside advances in relevant data and technologies.

## Author Contribution Statement

GI designed the manuscript. All authors contributed equally to writing the manuscript and approved the final version.

## Supplementary Material

Supplemental Figures

Supplemental Tables

## Figures and Tables

**Table 1.** List of functional markers for wheat breeding

Category	Trait	Gene	Chromosome	Assay	Reference
Abiotic stress tolerance	Drought tolerance	*1-FEH-w3*	6B	KASP	Rasheed *et al.* 2016
		*TaDreb-B1*	3B	PCR, KASP	Rasheed *et al.* 2016, Wei *et al.* 2009
	Preharvest sprouting	*TaMFT-A1*	3AS	PCR, CAPS, KASP	Nakamura *et al.* 2011, Rasheed *et al.* 2016
		*TaMKK3-A*	4A	CAPS	Torada *et al.* 2016
		*TaPHS1*	3A	KASP	Rasheed *et al.* 2016
		*TaSdr-B1*	2B	KASP	Rasheed *et al.* 2016
		*TaVp-B1*	3B	KASP	Rasheed *et al.* 2016
Adaptability	Heading time	*TaELF-B3*	1B	PCR	Mizuno *et al.* 2023
		*WPCL-A1*	3A	CAPS	Mizuno *et al.* 2016
		*WPCL-B1*	3B	PCR	Mizuno *et al.* 2016
		*WPCL-D1*	3D	CAPS	Mizuno *et al.* 2016
	Photoperiod sensitivity	*Ppd-A1*	2AS	PCR, KASP	Nishida *et al.* 2013, Rasheed *et al.* 2016
		*Ppd-B1*	2BS	PCR, KASP	Nishida *et al.* 2013, Rasheed *et al.* 2016
		*Ppd-D1*	2DS	PCR, KASP	Beales *et al.* 2007, Chen *et al.* 2013, Nishida *et al.* 2013, Rasheed *et al.* 2016
	Vernalization	*Vrn-A1*	5AL	PCR, KASP	Fu *et al.* 2005, Rasheed *et al.* 2016, Yan *et al.* 2004
		*Vrn-B1*	5BL	PCR, KASP	Chu *et al.* 2011, Fu *et al.* 2005, Rasheed *et al.* 2016
		*Vrn-D1*	5DL	PCR, KASP	Fu *et al.* 2005, Rasheed *et al.* 2016
		*Vrn-B3*	7BS	PCR	Yan *et al.* 2006
		*Vrn-D3*	7D	PCR	Chen *et al.* 2022
		*Vrn-D4*	5D	PCR	Kippes *et al.* 2015
Productivity	Reduced height	*Rht-B1*	4BS	PCR, KASP	Ellis *et al.* 2002, Rasheed *et al.* 2016
		*Rht-D1*	4DS	PCR, KASP	Ellis *et al.* 2002, Rasheed *et al.* 2016
		*Rht8*	2D	CAPS	Chai *et al.* 2022
	Yield	*GNI-A1*	2A	PCR	Sakuma *et al.* 2019
		*TaCKX-D1*	3D	KASP	Rasheed *et al.* 2016
		*TaCWI-4A*	4A	KASP	Rasheed *et al.* 2016
		*TaCWI-5D*	5D	KASP	Rasheed *et al.* 2016
		*TaCwi-A1*	2AL	PCR	Ma *et al.* 2012
		*TaGASR7-A1*	7A	KASP	Rasheed *et al.* 2016
		*TaGS-D1*	7D	KASP	Rasheed *et al.* 2016
		*TaMoc-A1*	7A	KASP	Rasheed *et al.* 2016
		*TaSus1-7A*	7A	PCR, KASP	Hou *et al.* 2014, Khalid *et al.* 2019
		*TaSus2-2B*	2B	PCR, KASP	Jiang *et al.* 2011, Rasheed *et al.* 2016
		*TaGW2-6A*	6AS	PCR	Su *et al.* 2011
		*TaTGW6-A1*	3A	KASP	Rasheed *et al.* 2016
Disease resistance	Fusarium head blight	*Fhb1*	3B	KASP	Rasheed *et al.* 2016
	Powdery mildew	*Pm3*	1AS	PCR	Tommasini *et al.* 2006
	Leaf rust	*Lr14a*	7BL	KASP	Rasheed *et al.* 2016
		*Lr21*	1DS	PCR, KASP	Rasheed *et al.* 2016, Talbert *et al.* 1994
		*Lr47*	7A	PCR, KASP	Helguera *et al.* 2000, Rasheed *et al.* 2016
		*Lr68*	7B	KASP	Rasheed *et al.* 2016
	Stem rust	*Sr2*	3B	KASP	Rasheed *et al.* 2016
		*Sr36*	2BS	KASP	Rasheed *et al.* 2016
	Stripe rust	*Yr15*	1B	KASP	Rasheed *et al.* 2016
		*Yr17*	2AS	PCR	Helguera *et al.* 2003
		*Yr36*	6B	KASP	Rasheed *et al.* 2016
	Leaf rust, stripe rust, and powdery mildew	*Lr34/Yr18/Pm38*	7DS	PCR, KASP	Lagudah *et al.* 2009, Rasheed *et al.* 2016
	Wheat blast	*Rmg8*	2BL	CAPS	Asuke *et al.* 2024
Quality	Amylose content	*SSII-A1*	7AS	PCR	Shimbata *et al.* 2005
		*SSII-B1*	7BS	PCR	Shimbata *et al.* 2005
		*SSII-D1*	7DS	PCR	Shimbata *et al.* 2005
		*Wx-A1*	7AS	PCR	Nakamura *et al.* 2002
		*Wx-B1*	4AL	PCR, KASP	Nakamura *et al.* 2002, Rasheed *et al.* 2016, Saito *et al.* 2009
		*Wx-D1*	7DS	PCR	Nakamura *et al.* 2002
	Grain hardness	*Pina-D1*	5DS	PCR, KASP	Ikeda *et al.* 2010, Rasheed *et al.* 2016
		*Pinb-B2*	7B	KASP	Rasheed *et al.* 2016
		*Pinb-D1*	5DS	PCR, KASP	Rasheed *et al.* 2016
	Grain protein content	*Gpc-B1*	6B	PCR, KASP	Distelfeld *et al.* 2006, Rasheed *et al.* 2016, Uauy *et al.* 2006
	HMW glutenin subunit	*Glu-A1*	1AL	PCR, KASP	Rasheed *et al.* 2016, Takata *et al.* 2008
		*Glu-B1*	1BL	PCR, KASP	Butow *et al.* 2004, Rasheed *et al.* 2016, Takata *et al.* 2008
		*Glu-D1*	1DL	PCR, KASP	Ahmad 2000, Ishikawa and Nakamura 2007, Rasheed *et al.* 2016
	LMW glutenin subunit	*Glu-A3*	1AS	PCR	Zhang *et al.* 2004
		*Glu-B3*	1BS	PCR	Ikeda *et al.* 2006, Maruyama-Funatsuki *et al.* 2005
	Lipoxygenase	*Lox-B1*	4B	PCR, KASP	Geng *et al.* 2012, Rasheed *et al.* 2016
	Peroxidase	*TaPod-A1*	3AL	KASP	Rasheed *et al.* 2016
	Phytoene synthase	*Psy-A1*	7AL	PCR, KASP	He *et al.* 2008, Rasheed *et al.* 2016
		*Psy-B1*	7BL	PCR, KASP	He *et al.* 2009, Rasheed *et al.* 2016
		*Psy-D1*	7D	KASP	Rasheed *et al.* 2016
	Polyphenol oxidase activity	*Ppo-A1*	2AL	PCR, KASP	He *et al.* 2007, Nakamaru *et al.* 2023, Nilthong *et al.* 2012, Rasheed *et al.* 2016
		*Ppo-D1*	2DL	PCR, KASP	He *et al.* 2007, Nakamaru *et al.* 2025, Rasheed *et al.* 2016
		*Ppo-A2*	2A	KASP	Beecher *et al.* 2012
		*Ppo-B2*	2B	KASP	Beecher *et al.* 2012
		*Ppo-D2*	2D	KASP	Beecher *et al.* 2012
	Zeta-carotene	*TaZds-A1*	2A	PCR, KASP	Dong *et al.* 2012, Rasheed *et al.* 2016
		*TaZds-D1*	2DL	PCR	Zhang *et al.* 2011
Others	Grain color	*Tamyb10-A1*	3AL	PCR	Himi *et al.* 2011
		*Tamyb10-B1*	3BL	PCR	Himi *et al.* 2011
		*Tamyb10-D1*	3DL	PCR	Himi *et al.* 2011
	Rye translocation	1RS:1BL	1BS	PCR, KASP	Francis *et al.* 1995, Rasheed *et al.* 2016
		1RS:1AL	1AS	KASP	Rasheed *et al.* 2016

**Table 2.** Wheat Genome databases

Website	URL	Comments
URGI	https://wheat-urgi.versailles.inra.fr/	IWGSC RefSeq RefSeq 2.1
CAU	https://wheat.cau.edu.cn/CS-CAU/	CS-CAU
KOMUGI	https://shigen.nig.ac.jp/wheat/komugi/genome/download.jsp	Fielder
EnsemblPlants	https://plants.ensembl.org/Triticum_aestivum/Info/Index	10+ genome data
GrainGenes	https://wheat.pw.usda.gov/	Portal site
IPK	https://galaxy-web.ipk-gatersleben.de	Portal site
PlantGARDEN	https://plantgarden.jp/en/list/t4565	Portal site

**Table 3.** List of wheat SNP arrays on array size, technologies and references

Array name	Size	Technology	Reference
Illumina Wheat 9K iSelect SNP array	9K	Illumina Infinium BeadChip	Cavanagh *et al.* 2013
Illumina Wheat 90K iSelect SNP genotyping array	90K	Illumina Infinium BeadChip	Wang *et al.* 2014
Axiom^®^ HD Wheat genotyping (820K) array	820K	Affymetrix Axiom	Winfield *et al.* 2016
Wheat Breeders’ 35K Axiom array	35K	Affymetrix Axiom	Allen *et al.* 2017
Axiom^®^ Wheat 660K SNP array	660K	Affymetrix Axiom	Cui *et al.* 2017
TaBW280K SNP array	280K	Affymetrix Axiom	Rimbert *et al.* 2018
TaBW410K SNP array	410K	Affymetrix Axiom	Kitt *et al.* 2021
TaNG v1.1 arrays	43K	Affymetrix Axiom	Burridge *et al.* 2024

**Table 4.** Various methods for obtaining wheat genomic information and their characteristics

Type	Subtype	Representative method	Description	Preliminary information	DNA quality requried	No. of simultaneously analyzed samples	Experimental cost	Cost per sample [Yen]*	Data analysis cost	Note
Whole genome sequencing	Deep	WGS	In depth sequence, 10–20x coverage	Not required	High	Low	High	200,000–300,000	Very high	Suitable for sequence and structual variants discovery
	Skim	Skim seq	Skim sequence, 1–2x coverage	Requried	High	High	Middle	20,000–30,000	Middle	Intensive imputation required
Partial genome sequencing	Random	RAD-seq	Genomic DNA is treated with restriction enzymes, and then only the area around the recognition site is sequenced.	Not required	High	High	Low	2000–5000	High	Intensive imputation required. Polymorphisms may occur less frequently in closely related material or have a chromosomal bias
		GRAS-Di, MIG-seq	Sequence of amplified products using random primers (GRAS-Di) or primers with microsatellite motifs (MIG-seq)	Not required	Low	High	Low	2000–5000	Middle	Polymorphisms may occur less frequently in closely related material or have a chromosomal bias
	Targeted	Amplicon seq	Sequence of amplified fragments using primers that spanning known polymorphic sites	Requried	Low	High	Low	1000–3000	Low	
		Seq Cap	Enrich target sequences using probes by hybridization and sequence	Requried	Middle	Middle	Middle	40,000–60,000	Middle	Suitable for allele mining
SNP array	High density	HD array	Probes containing known polymorphisms are arrayed on slides and SNPs are detected by fluorescence. More than 100K probes.	Requried	Middle	Middle	Middle	5000–10,000	Low	
	Low density	LD array	Probes containing known polymorphisms are arrayed on slides and SNPs are detected by fluorescence. Less than 100K probes.	Requried	Middle	High	Low	3000–5,000	Low	

* Costs are approximate. They vary depending on the number of samples to be analyzed at any one time, and whether the experiments and analyses are carried out by the institution itself or outsourced.

**Table 5.** Recommended genome-wide genotyping platform for wheat by application and material

Objective	Application	Breeding materials	Novel materials
Genetic analysis	QTL analysis using bi-parental population	Amplicon seq Skim seq	MIG-seq GRAS-Di
	Diversity analysis	HD/LD array Amplicon seq	GBS RAD-seq HD array
	GWAS	HD/LD array	WGS HD array
Breeding selection	Background selection	Amplicon seq	
	Genomic prediction	LD array Amplicon seq	
