# Supplementary material for: Current status and future perspective of genomics-assisted breeding in wheat (Triticum aestivum L.)
Source: Breed Sci. 2026 Feb 21;76(1):48–62. doi: 10.1270/jsbbs.25059 (PMC13437168; doi:10.1270/jsbbs.25059)
Supplement: Supplementary file 1 [file 76_048_s1.pdf]

doi: 10.1270/jsbbs.25059

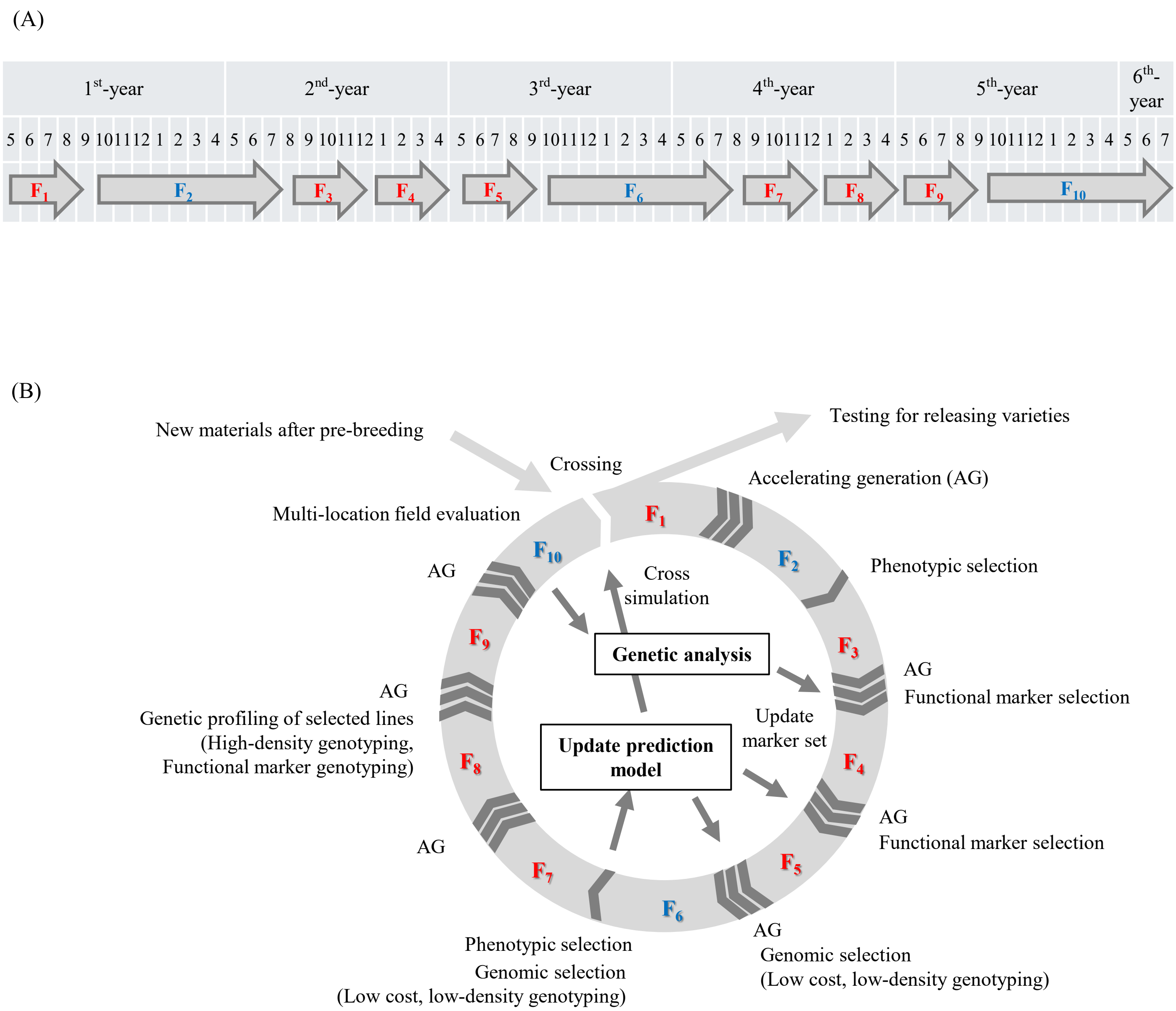

Supplemental Fig. 1. A schematic of the proposed breeding process "Breeding Ring" in an area where the wheat growing season is from eight to ten months, e.g. northeastern Japan. (A) Cultivation calendar from F_1 to F_{10}. (B) Work items carried out at each generation.

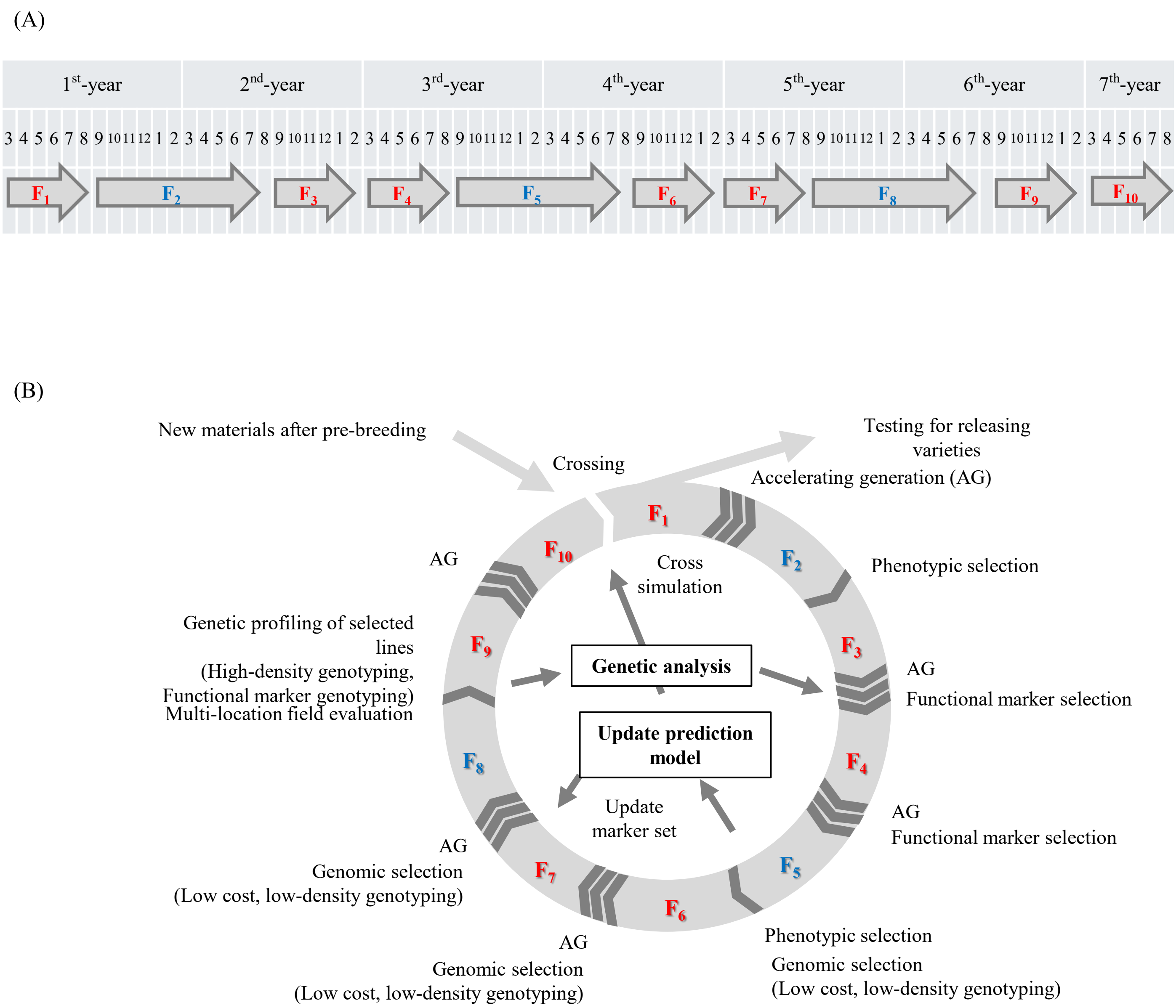

Supplemental Fig. 2. A schematic of the proposed breeding process “Breeding Ring” in an area where wheat growing season is ten months or longer. (A) Cultivation calendar from F_1 to F_{10}. (B) Work items carried out at each generation.
