# Supplementary material for: Current status and future perspective of genomics-assisted breeding in wheat (Triticum aestivum L.)
Source: Breed Sci. 2026 Feb 21;76(1):48–62. doi: 10.1270/jsbbs.25059 (PMC13437168; doi:10.1270/jsbbs.25059)
Supplement: Supplementary file 2 [file 76_048_s2.pdf]

doi: 10.1270/jsbbs.25059

Supplemental Table 1. Difference between the same version of wheat cultivar 'Chinese Spring' genomes.

		URGI (RefSeq v. 2.1)	GenBank (RefSeq v.2.1)*	CS-CAU	CS-IAAS
Total length		14577412364	14566896347	14456220132	14509208007
# of seqs		22	91589	25	21
Chromosome lengths					
	Chr1A	598660471	598660471	602244791	603818960
	Chr1B	700547350	700547350	716849971	723777933
	Chr1D	498638509	498638509	504218279	505466611
	Chr2A	787782082	787782082	792376954	788135348
	Chr2B	812755788	812755788	822365236	822617018
	Chr2D	656544405	656544405	659355969	661150927
	Chr3A	754128162	754128162	759722960	759363255
	Chr3B	851934019	851934019	867044581	873778448
	Chr3D	619618552	619618552	633415760	635914663
	Chr4A	754227511	754227511	760279503	768317091
	Chr4B	673810255	673810255	707212945	696896727
	Chr4D	518332611	518332611	527505115	529961705
	Chr5A	713360525	713360525	719314300	722258891
	Chr5B	714805278	714697677	733108632	742820188
	Chr5D	569951140	569951140	580278433	581969875
	Chr6A	622669697	622669697	626338564	630957784
	Chr6B	731188232	731188232	747346816	756690666
	Chr6D	495380293	495380293	503123466	507676638
	Chr7A	744491536	744491536	750417019	752191036
	Chr7B	764081788	764072961	778960951	785289892
	Chr7D	642921167	642921167	660265859	660154351
Total length of 21 chromosomes		14225829371	14225712943	14451746104	14509208007
Gene number**					
	Chr1A	5137	5730	5942	6959
	Chr1B	5524	6226	7115	8132
	Chr1D	5315	5891	6291	6880
	Chr2A	6805	7451	8332	9140
	Chr2B	7181	8106	9538	10385
	Chr2D	6988	7722	8793	10019
	Chr3A	6306	6959	7261	8536
	Chr3B	7083	8057	9313	10567
	Chr3D	6545	7057	7765	8734
	Chr4A	5762	6245	7220	8269
	Chr4B	4666	5536	6057	6678
	Chr4D	4428	5189	5464	6010
	Chr5A	6354	6915	7276	8534
	Chr5B	6538	7460	8044	9236
	Chr5D	6570	7371	7528	8982
	Chr6A	4863	5177	5810	6815
	Chr6B	5382	6546	7464	8224
	Chr6D	4746	5567	6088	6753
	Chr7A	6450	7011	8028	8875
	Chr7B	5825	6867	7663	8434
	Chr7D	6337	7300	8244	8781
	Contigs/scaffolds	1439	2045	52	0
	Organelle (mitochondrion)		14		
Total gene number		126244	142442	155288	174943

*GCF_018294505.1

**We count only high confidence and validated genes for URGI.

Supplemental Table 2. Various online genome analysis and dat]a visualization tools

Name	Description	Reference	URL (example site)
BLAST	sequence homology search	Altschul *et al.* 1990	https://blast.ncbi.nlm.nih.gov/Blast.cgi (NCBI)
CD-Search	Functional domain search	Marchler-Bauer and Bryant 2006	https://www.ncbi.nlm.nih.gov/Structure/cdd/wrpsb.cgi (NCBI)
InterProScan	Functional domain search	Quevillon *et al.* 2005	https://www.ebi.ac.uk/interpro/search/sequence/ (EBI)
GOslim	GO analysis and enrichment analysis	McCarthy *et al.* 2006	https://agbase.arizona.edu/ (Agbase)
OrthoVenn3	Ortholog analysis	Sun, Lu *et al.* 2023	https://orthovenn3.bioinfotoolkits.net/
DeepLoc - 2.1	Prediction of subcellular localization	Ødum *et al.* 2024	https://services.healthtech.dtu.dk/services/DeepLoc-2.1/ (DTU Health Tech)
Phyre2.2	Protein structure prediction	Powell *et al.* 2025	https://www.sbg.bio.ic.ac.uk/~phyre2/html/page.cgi?id=index
TBtools-II	Platform for biological big-data mining	Chen *et al.* 2023	https://github.com/CJ-Chen/TBtools-II
JBrowse2	Genome browser	Diesh et al. 2023	https://jbrowse.org/jb2/
IGV	Integrative Genomics Viewer	Robinson et al. 2011	https://igv.org/
TASUKE+	Multiple genome browser	Kumagai *et al.* 2019	https://tasuke.dna.affrc.go.jp/
MEGA	Molecular Evolutionary Genetics Analysis	Kumar *et al.* 2024	https://www.megasoftware.net/

Supplemental Literature Cited

Chen, C., Y. Wu, J. Li, X. Wang, Z. Zeng, J. Xu, Y. Liu, J. Feng, H. Chen, Y. He *et al* . (2023) TBtools-II: A “one for all, all for one” bioinformatics platform for biological big-data mining. Mol Plant 16: P1733–P1742.

Diesh, C., G. Stevens, P. Xie, T. Martinez, E. Hershberg, A. Leung, E. Guo, S. Dider, J. Zhang, C. Bridge *et al.* (2023) JBrowse 2: a modular genome browser with views of synteny and structural variation. Genome Biol 24: 74.

Kumar, S., G. Stecher, M. Suleski, M. Sanderford, S. Sharma and K. Tamura (2024) MEGA12: molecular evolutionary genetic analysis version 12 for adaptive and green computing. Mol Biol Evol 41: 1–9.

Marchler-Bauer, A. and S.H. Bryant (2006) CD-Search: protein domain annotations on the fly. Nucleic Acids Res 32 (Web Server issue): W327–W331.

McCarthy, F., N. Wang, G. Magee, B. Nanduri, M. Lawrence, E. Camon, D. Barrell, D. Hill, M. Dolan, W. Williams *et al.* (2006) AgBase: a functional genomics resource for agriculture. BMC Genomics 7: 229.

Ødum, M.T., F. Teufel, V. Thumuluri, J.J. Almagro Armenteros, A.R. Johansen, O. Winther and H. Nielsen (2024) DeepLoc 2.1: multi-label membrane protein type prediction using protein language models. Nucleic Acids Res 52 (W1): W215–W220.

Powell, H.R., S.A. Islam, A. David and M.J.E. Sternberg (2025) Phyre2.2: a community resource for template-based protein structure prediction. J Mol Biol 437: 168960.

Quevillon, E., V. Silventoinen, S. Pillai, N. Harte, N. Mulder, R. Apweiler and R. Lopez (2005) InterProScan: protein domains identifier. Nucleic Acids Res 33 (Web Server issue): W116–W120.

Robinson, J., H. Thorvaldsdóttir, W. Winckler, M. Guttman, E. Lander, G. Getz and J. Mesirov (2011) Integrative genomics viewer. Nat Biotechnol 29: 24–26.
